# Investigation of the potential of *Glycyrrhiza glabra* as a bioavailability enhancer of Vitamin B12

**DOI:** 10.3389/fnut.2022.1038902

**Published:** 2022-10-28

**Authors:** Priyanka Sharma, Priyanka Pathak, Vidushi Tyagi, Feroz Khan, Karuna Shanker, Mahendra Pandurang Darokar, Anirban Pal

**Affiliations:** ^1^Bioprospection and Product Development, CSIR-Central Institute of Medicinal and Aromatic Plants, Lucknow, Uttar Pradesh, India; ^2^Computational Biology, CSIR-Central Institute of Medicinal and Aromatic Plants, Lucknow, Uttar Pradesh, India; ^3^Analytical Chemistry, CSIR-Central Institute of Medicinal and Aromatic Plants, Lucknow, Uttar Pradesh, India

**Keywords:** Vitamin B12, bioavailability enhancer, *Glycyrrhiza glabra*, intestinal absorption, Caco-2, everted gut sac, pharmacokinetics

## Abstract

Vitamin B12 deficiency is prevalent among individuals globally. Inadequate consumption of B12 rich diet and low bioavailability (due to diet based/physiological factors) are linked to the deficiency of Vitamin B12 inside the body. Bioavailability enhancers augment the bioavailability of an ingested substance (drug/nutrient) thus increasing their concentration inside the body and maximizing their therapeutic benefits. In traditional medicine, Licorice (*Glycyrrhiza glabra*) finds utility in the treatment of various health conditions. Thus, the present study aimed to examine the potential of ethanolic extract obtained from *G. glabra* roots to enhance the bioavailability of Vitamin B12. The effect of ethanolic extract of *G. glabra* (GgEtOH) on intestinal absorption enhancement of B12 was assessed *in vitro* on Caco-2 and *ex-vivo* everted gut sac models. The influence of extract on the pharmacokinetics of Vitamin B12 was determined *in vivo* in Swiss albino mice. GgEtOH significantly enhanced the permeation (Papp) of B12 by 2-5 fold *in vitro* (25, 50, and 100 μg/ml concentrations) and *ex-vivo* (250 and 500 μg/ml concentrations). The pharmacokinetic parameters of B12 such as Cmax, AUC, Tmax, etc. were also significantly elevated *in vivo* upon oral administration of B12 (1 mg/kg dose) in combination with GgEtOH (100 and 1,000 mg/kg dose). These preliminary findings indicate that the ethanolic extract of *G. glabra* is capable of enhancing the bioavailability of Vitamin B12. To the best of our knowledge, this is the first report to demonstrate herbal extract-mediated enhancement of Vitamin B12 bioavailability through *in vitro, ex vivo*, and *in vivo* assays.

## Introduction

Micronutrients are nutrient elements required in trace amounts and are components of crucial metabolic processes occurring inside the body (1). Among various micronutrients, Vitamin B12 holds significant importance from health perspective. Vitamin B12 belongs to the “corrinoid” group of compounds that contain a corrin macrocycle (2). Functional forms of Vitamin B12 include Cyanocobalamin, Methylcobalamin, and Hydroxocobalamin. Severe clinical deficiency of B12 in the body gets expressed in form of megaloblastic anemia (due to defect in DNA synthesis during RBCs formation), neuropathies, and cardiovascular abnormalities (occurring due to increased concentration of methylmalonic acid (MMA) and homocysteine (Hcys) in the blood (3–5). Malnutrition ensuing from deficiency of vitamin B12 is widespread across the globe. B12 deficiency in the body occurs as a consequence of reduced dietary intake and reduced bioavailability/malabsorption caused by various factors such as pernicious anemia, congenital defects in proteins participating in Vitamin B12 absorption, bacterial overgrowth, inflammatory bowel disease (IBD), gastro-intestinal surgeries, and various drugs that interact with B12 and inhibit its absorption (6). Moreover, individuals surviving on vegan diet are more prone to developing B12 deficiency than those who consume animal-derived food that contains a high proportion of bioavailable B12 (7).

Low bioavailability is often associated with reduced micronutrient status in the body. Bioenhancers are the agents/entities that augment the bioavailability of drugs/nutrients upon co-administration (8). The bioavailability of a nutrient can be defined as the amount of ingested nutrient that is absorbed and made available in the body to participate in different physiological processes (9). Nutrient bioavailability is affected by three parameters like availability of nutrients in the intestinal lumen for absorption (bioaccessibility), intestinal membrane permeation (absorption) followed by transport into the blood for systemic circulation and utilization in the body (10). Several plant-based molecules have been recognized as natural bioenhancers of drugs which include Piperine, Ginger, Drumstick pod, Licorice, Black cumin, Caraway, Garlic, Quercetin, Sinomenine, Aloe vera, etc. However, only a few of them have been reported to enhance the bioavailability of some micronutrients upon oral supplementation in human subjects and animal models (11, 12).

*Glycyrrhiza glabra* also known as licorice is a herb belonging to the Leguminosae family with numerous species distributed in various geographical regions worldwide. This herb has vast therapeutic applications owing to the presence of numerous phytomolecules associated with diverse biological effects that are beneficial to health (13). Hence, the following study was undertaken to assess the effect of ethanolic extract of *G. glabra* on promoting the bioavailability of Vitamin B12. Since, the major site of absorption of ingested micronutrients is the small intestine, bioavailability of Vitamin B12 is affected due to inadequate absorption under various conditions leading to deficiency. Bioavailability enhancers are recognized for their role in facilitating the intestinal permeation of target molecules to enhance their oral bioavailability. Here, we hypothesize that the ethanolic extract of *G. glabra* (GgEtOH) enhances oral bioavailability of Vitamin B12 by promoting its intestinal absorption. To validate our hypothesis, we carried out different assays with the ethanolic extract of *G. glabra* (GgEtOH) which included *in vitro* Caco-2 and *ex vivo* everted gut sac assays to assess B12 intestinal absorption enhancement by GgEtOH followed by *in vivo* assay (B12 pharmacokinetics study in mice in presence of GgEtOH).

## Materials and methods

Cyanocobalamin, HBSS salt (with phenol red), Tween-80, sodium pyruvate, glucose, sodium chloride, sodium bicarbonate, calcium chloride, sodium dihydrogen phosphate, potassium dihydrogen phosphate, magnesium chloride, potassium chloride, dimethylsulfoxide (DMSO), EDTA were purchased from Sigma-Aldrich (St. Louis, MO, United States), Minimum Essential Medium Eagle’s (MEM), trypsin, Penicillin Streptomycin solution (100×), Amphotericin B solution (250 μg/ml), HBSS (w/o phenol red), Fetal Bovine Serum (FBS) were purchased from Gibco (Grand Island, NY, United States), 12 mm transwell plate (with 0.4 μm pore polyester membrane inserts) was purchased from Corning Costar (USA), formic acid, perchloric acid, methanol, and acetonitrile (HPLC grade) were purchased from Merck- Millipore India.

### Preparation of ethanolic extract of *Glycyrrhiza glabra*

For the preparation of ethanolic extract of *G. glabra*, mature roots of the plant were powdered and mixed with 95% alcohol (1:10 ratio) under continuous agitation at 230 rpm at 40°C for 7 h. The extract was then subjected to vacuum concentration. For HPLC analysis, the extract was dissolved in methanol at a concentration of 1 mg/ml and filtered through a 0.45 μm syringe filter (14).

### HPLC analysis of ethanolic extract of *Glycyrrhiza glabra*

HPLC analysis was carried out on Waters Alliance 2695 separation module equipped with 2998 photodiode array detector and Phenomenex C18 Luna^®^ column (5 μm; 250 mm × 4.6 mm). Standards were gifted by Dr. R S Bhakuni. The separation of six markers namely (a) Liquiritigenin (LTG), (b) Isoliquiritigenin (ISL), (c) Formononetin (F), (d) Glycyrhyzzic acid (GA), (e) Glabridin (GLB), and (f) Glycyrrhetinic acid (GTA) was optimized using the mobile phase of (a) water (0.1% formic acid) and (b) acetonitrile (0.1% formic acid) with the given gradient elution using a C18 column (5 μm; 250 mm × 4.6 mm) at a flow rate of 1 ml/min. The column eluent was monitored at 254 nm (14, 15).

**Table:** 

Time (min)	Flow rate (ml/min)	%B
Initial	1.0	15
27.0	1.0	45
37.5	1.0	80
50.0	1.0	80
51	1.0	15

### Cell culture

Caco-2 cell line was procured from National Centre for Cell Sciences (NCCS), Pune (India). The cell line was routinely maintained in MEM (low glucose) growth medium containing 20% FBS, 1% L-glutamine, 1 mM sodium pyruvate, 1× penicillin-streptomycin, and 1× (2.5 μg/ml) amphotericin B. Cells were grown in T-25 flasks at 37°C, 5% CO_2_, 95% humidity with growth medium being changed three times a week and sub-cultured upon reaching 80% confluence with Trypsin EDTA solution (0.25% Trypsin, 53 mM EDTA in Hanks Balanced Salt Solution (HBSS with phenol red, pH 7.4).

For *in vitro* Vitamin B12 permeability experiments, Caco-2 cells were seeded at a density of 1,00,000 cells in the apical chamber (AP) of Polyethylene Terephthalate (PET) inserts of 12 well transwell plates. A total of 500 and 1,000 μl growth medium was added to the apical (AP) and basolateral (BL) chambers of each transwell, respectively. Growth medium was changed in both AP and BL compartments on alternate days and cells were allowed to grow for 14 days until the formation of tight differentiated monolayers in the AP chambers of inserts. The differentiation status of monolayers was assessed by the measurement of trans-epithelial electrical resistance (TEER) by EVOM^2^ Voltohmeter (WPI, USA) every alternate day. TEER was calculated as Actual TEER = TEER (cell bearing insert) − TEER (blank insert) × area of insert (1.12 cm^2^). The differentiated Caco-2 monolayers with TEER values ranging from 3,000 to 4,000 Ω.cm^2^ were further considered for the *in vitro* study (16, 17).

### Vitamin B12 permeability assay in Caco-2 model

Well-differentiated Caco-2 monolayers with TEER values stated above were employed in *in vitro* study. On the day of the experiment, the growth medium in Caco-2 monolayer bearing transwells was replaced with HBSS (w/o phenol red, with 25 mM HEPES pH 7.4). Monolayers were washed once with HBSS (500 μl in AP and 1,200 μl in BL compartment) followed by equilibration i.e., incubation in HBSS for 30 min at 37°C in a shaker incubator. After completion of the equilibration step, the *in vitro* assay was initiated. Stock solutions of Vitamin B12 (Cyanocobalamin) and GgEtOH were prepared in Milli-Q water and DMSO, respectively which were diluted to final working concentrations in HBSS. To the apical chamber bearing Caco-2 monolayers, B12 was added at a concentration of 200 μg/ml (17) and GgEtOH was added at 25, 50, and 100 μg/ml concentration in HBSS (500 μl) whereas basolateral chamber contained only HBSS (1,200 μl). Apical chambers containing only B12 and the combination of B12 + GgEtOH served as control and treatment groups, respectively. The transwell plate was then incubated at 37°C with constant shaking at 50 rpm (Biosan ES-20 orbital shaker incubator). Samples (200 μl) were withdrawn from basolateral chamber after 30 min intervals for 240 min followed by the addition of 200 μl of fresh HBSS to the BL compartment. After assay completion, B12 was quantitated in basolateral samples (collected at various time intervals) spectrophotometrically by measuring absorbance at 360 nm using a microplate reader (FLUOstar Omega). The concentration of Vitamin B12 in samples was deduced from the standard curve followed by the determination of Papp. Two independent experiments were performed with duplicates comprising fully differentiated Caco-2 monolayers treated with B12 only (200 μg/ml) and combination of B12+GgEtOH (25, 50,100 μg/ml).

Apparent permeability coefficient, Papp (cm/s) was calculated from the following equation:


Papp=dQ/dt*1/A.C0

where, dQ/dt (μg/s) is the flux rate, A (cm^2^) is the surface area of the apical chamber bearing cell monolayer, and C0 (μg/ml) is the initial B12 concentration (apical chamber).

Fold enhancement (FE) was further calculated as the ratio of Papp (B12+GgEtOH) and Papp (B12 alone).


FE=Papp⁢(B12+GgEtOH)/Papp⁢(B12⁢alone)

### *Ex vivo* assay for intestinal permeation of Vitamin B12

Everted gut sac assay was performed to determine intestinal permeation enhancement of Vitamin B12 in presence of GgEtOH utilizing Swiss albino mice weighing 25–30 g. Everted gut sacs were prepared following the protocol described (18). Mice were sacrificed by cervical dislocation and the small intestine was isolated in oxygenated Krebs Ringer Buffer (KRB) (Glucose 7.78 mM, NaCl 133 mM, KCl 4.56 mM, NaH_2_PO4 1.5 mM, MgCl_2_ 0.20 mM, NaHCO_3_16 mM, CaCl_2_3.33 mM, pH 7.4) (19). 10 cm portion from the ileocecal junction (corresponding to the ileum) was separated from the rest of the small intestine. The ileum was flushed with cold normal saline to remove its contents. Ileum was then cut into 4 cm sized segments and gently everted on thin plastic capillary tubing. One end of each everted segment was tied with cotton thread and 500 μl oxygenated KRB was filled through the tubing from another end after which it was tied to create everted gut sacs. Prepared everted gut sacs were initially incubated in oxygenated KRB at 37°C for 10 min. Sacs were then transferred to tubes containing 2 ml oxygenated KRB with added B12 (Cyanocobalamin) at a concentration of 100 μg/ml and GgEtOH extract at 250 and 500 μg/ml concentrations and incubated at 37° for 2 h with constant aeration and shaking at 100 rpm. Sacs incubated with B12 alone served as the control/untreated group whereas sacs incubated with B12+GgEtOH were included in treatment groups. After 30, 60, 90, and 120 min sacs were cut open, serosal fluid collected in 2 ml tubes and short spun for 5 min to separate the debris from the fluid. Hundred microliter of serosal fluid was transferred to 96 wells plate and Vitamin B12 was quantitated from the standard curve by measuring absorbance at 360 nm. Area of each sac was calculated considering it a cylinder (length 4 cm, width/diameter 0.5 cm, radius 0.25 cm) (20). Two experiments were performed, each with two replicates comprising everted gut sacs treated with B12 only (100 μg/ml) and combination of B12+GgEtOH (250 and 500 μg/ml). Apparent permeability coefficient (Papp) was then calculated by the following equation:


Papp=d⁢Q/d⁢t*1/A.C0

where, dQ/dt (μg/s) is the flux rate, A (cm^2^) is the surface area of the everted gut sac and C0 (μg/ml) is the initial B12 concentration (mucosal compartment).


Fold⁢enhancement⁢(FE)=Papp⁢(B12+GgEtOH)/Papp⁢(B12⁢alone)

### *In vivo* Vitamin B12 pharmacokinetics study

The pharmacokinetic study of Vitamin B12 was carried out in Swiss albino mice (male), bred and maintained under standard conditions and fed with ATNT 1324-Maintenance Diet pellets. Experiments were conducted under protocol (CIMAP/IAEC/2019-21/06) approved by the Institutional Animal Ethics Committee under the aegis of the Committee for the Purpose of Control and Supervision of Experiments on Animals (CPCSEA), Government of India.

Swiss albino mice (weighing 25 g) were divided into 12 experimental groups with five mice per group based on different doses (B12 and GgEtOH) and different time intervals (15, 30, 60,120 min). The experimental groups were as follows:

B12 alone (1 mg/kg) (15, 30, 60, 120 min)— 20 animals

B12 (1 mg/kg) + GgEtOH (100 mg/kg) (15, 30, 60, 120 min)—20 animals

B12 (1 mg/kg) + GgEtOH (1000 mg/kg) (15, 30, 60, 120 min)—20 animals

Prior experiment, animals were fasted for 18–24 h with access to water only. Animals were orally administered Cyanocobalamin alone and in combination with GgEtOH extract at doses indicated. For oral administration, GgEtOH extract was initially solubilized in 1% Tween 80 and then the final volume was made up with 0.5% CMC in Milli-Q water. B12 (Cyanocobalamin) solution was also prepared similarly. After defined time intervals (15, 30, 60, and 120 min), 500 μl blood was withdrawn from the orbital plexus of animals and collected in 2 ml tubes containing 50 μl ACD (Acetate Citrate Dextrose) solution (21, 22).

### Vitamin B12 detection by HPLC

Blood samples were processed for the quantitation of Vitamin B12 by the HPLC method as described elsewhere (21). The processed samples were then transferred to a reversed-phase HPLC system (Waters) equipped with Bridge C18 5 μm column (4.6 mm × 250 mm). The mobile phase consisted of methanol: phosphate buffer (20 mM, pH 3.5) (25:75) with a flow rate of 0.6 ml/min. Vitamin B12 was detected at a wavelength of 360 nm. Pharmacokinetic parameters were evaluated by PK Solver 2.0 software.

### Statistical analysis

Results are expressed as mean ± SD. Statistical analysis was performed on Graph pad prism software (version 5.0). Significance between control (B12 alone) and treatment (B12+GgEtOH) groups was determined by ANOVA followed by Dunnett’s *post-hoc* test. *p* < 0.05 was considered significant.

## Results

### HPLC profile of ethanolic extract of *Glycyrrhiza glabra*

**FIGURE 1:**
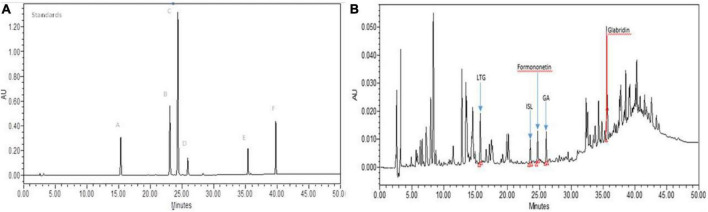
**(A)** Representative HPLC chromatogram of the standard mix (1.0 mg/ml) of (A) Liquiritigenin (LTG), (B) Isoliquiritigenin (ISL), (C) Formononetin, (D) Glycyrrhizic acid (GA), (E) Glabridin (GLB), and (F) Glycyrrhetinic acid (GTA) at 254 nm. **(B)** HPLC chromatogram of Ethanolic extract of *Glycyrrhiza glabra* (GgEtOH) at 254 nm. LTG—0.65%, ISL—0.17%, GA—0.69%, GLB—1.65%, F—0.12%.

Figure 1A represents the chromatogram comprising standard markers separated by the HPLC protocol described. HPLC fingerprint of GgEtOH has been shown in Figure 1B. The content of marker compounds detected in GgEtOH was LTG—0.65%, ISL—0.17%, F—0.12%, GA—0.69%, and GLB—1.65%.

### *In vitro* Vitamin B12 permeability assay in Caco-2 model

**FIGURE 2:**
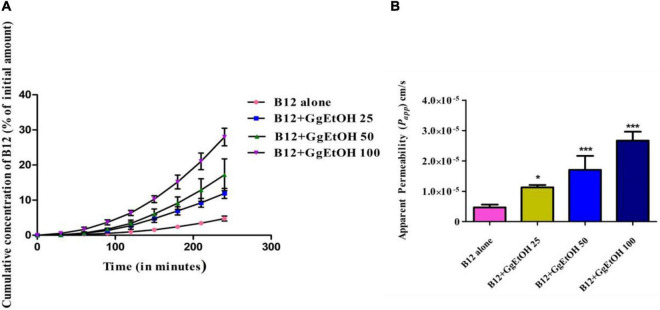
**(A)** Cumulative amount of B12 transport with time (expressed as % of initial concentration) across Caco-2 monolayers. Each point represents mean ± SD (*n* = 4). **(B)** Effect of GgEtOH on permeability enhancement of Vitamin B12 in *in vitro* Caco-2 assay. B12 alone—Caco 2 monolayers treated with 200 μg/ml Cyanocobalamin only, B12+GgEtOH 25, B12+GgEtOH 50, B12+GgEtOH 100—Caco 2 monolayers treated with combination of Cyanocobalamin 200 μg/ml and GgEtOH at concentrations 25, 50, and 100 μg/ml, respectively. Data presented as mean ± SD (*n* = 4) *p < 0.05, **p < 0.01, ***p < 0.001 with respect to B12 alone.

Vitamin B12 permeation study was carried out *in vitro* in the Caco-2 model to evaluate the effect of GgEtOH on enhancing intestinal absorption of B12. In presence of GgEtOH, the rate of transport of B12 across Caco-2 monolayers was found to increase gradually with time in a dose-dependent manner (Figure 2A). At 25, 50, and 100 μg/ml GgEtOH concentration, permeation of B12 was significantly enhanced compared to control as indicated by an increase in *Papp* values of B12 in Caco-2 monolayers treated with B12+GgEtOH (25, 50, 100) combination (Figure 2B). GgEtOH at 25, 50, and 100 μg/ml concentration enhanced B12 absorption by 2.47, 3.68, and 5.9 fold respectively thereby demonstrating an effect on enhancing intestinal membrane permeation (Supplementary Table 1).

### *Ex vivo* Vitamin B12 permeability study

**FIGURE 3:**
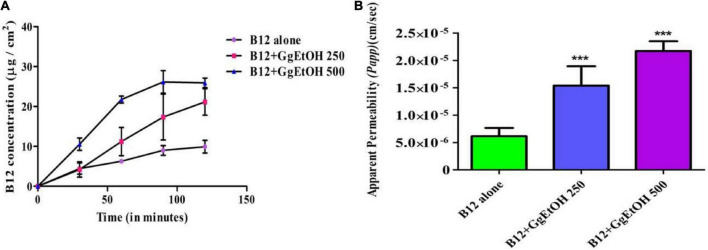
**(A)** Concentration vs. time profile of B12 transport across gut sacs (from mucosal to serosal compartment). Each point represents mean ± SD (n = 4). **(B)** Effect of GgEtOH on intestinal permeation enhancement of Vitamin B12 in everted gut sac assay. B12 alone (sacs incubated with Cyanocobalamin only at concentration of 100 μg/ml), B12+GgEtOH 250, B12+GgEtOH 500 [Sacs incubated with combination of Cyanocobalamin (100 μg/ml) and GgEtOH at 250 and 500 μg/ml concentration, respectively]. **p < 0.01, ***p < 0.001 with respect to B12 alone (*n* = 4).

Everted gut sac assay was performed with the ileum segments of mouse small intestine to assess the possible effect of GgEtOH on enhancing the permeation of B12 through the intestinal membrane. Sacs incubated with Cyanocobalamin only served as the control group. GgEtOH enhanced absorptive transport of B12 as demonstrated by an increased rate of accumulation of B12 in gut sacs incubated with a combination of B12+GgEtOH than B12 alone (Figure 3A). As shown in Figure 3B, increased apparent permeability *(Papp)* values of B12 were obtained for sacs incubated with B12+GgEtOH (250 and 500 μg/ml) as compared to B12 alone. GgEtOH extract enhanced intestinal permeation of B12 by 2.6 and 3.7 fold at 250 and 500 μg/ml concentration, respectively during the 2-h experimental duration (Supplementary Table 2). It can thus be considered that the extract is capable of enhancing the absorption of B12 through the small intestine.

### *In vivo* Vitamin B12 pharmacokinetics study

**FIGURE 4:**
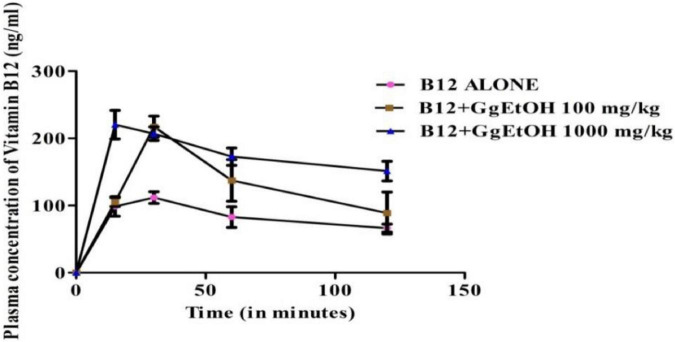
Plasma concentration vs. time profile of Vitamin B12 during *in vivo* B12 pharmacokinetics study in presence of GgEtOH. Data presented as mean ± SD (*n* = 5).

The pharmacokinetics study of Vitamin B12 was performed in Swiss albino mice to investigate the effect of GgEtOH on the bioavailability enhancement of B12. Vitamin B12 (Cyanocobalamin) at a dose of 1 mg/kg body weight, alone and in combination with GgEtOH extract (100 and 1,000 mg/kg dose) was administered to animals through oral route. B12 content was determined in blood plasma samples collected at 15, 30, 60, and 120 min post-dosing by HPLC. As shown in Figure 4, the plasma concentration of Vitamin B12 increased in mice treated with B12+GgEtOH 100 mg/kg and B12+GgEtOH 1,000 mg/kg compared to mice treated with B12 only. Further, the pharmacokinetic profile of Vitamin B12 was significantly enhanced in presence of extract. Although, GgEtOH at 100 mg/kg dose significantly increased the Cmax and AUC, it did not contribute to the increment in t_1/2_, Tmax, AUC _0–inf_ and MRT of B12. However, GgEtOH at 1,000 mg/kg dose caused significant enhancement of Cmax and AUC compared to the control group. At 1,000 mg/kg dose of extract, although not significant, but an increase in half-life (t_1/2_) and mean residence time (MRT) of B12 was also observed. In B12+GgEtOH 1,000 mg/kg treated group, the Tmax of B12 was significantly improved and was attained in lesser duration (18.75 min) than B12 alone treated group (26.25 min) (Table 1). The results indicated that 1,000 mg/kg dose of the extract led to the enhanced absorption of B12 than 100 mg/kg dose. Overall, GgEtOH improved the bioavailability of Vitamin B12 as evidenced by pharmacokinetic study.

**TABLE 1:** Pharmacokinetic parameters of Vitamin B12 during *in vivo* study.

	B12 alone (1mg/kg)	B12+GgEtOH (100 mg/kg)	B12+GgEtOH (1000 mg/kg)
t1/2	121.27 ± 34.74	89.21 ± 54.58	208.65 ± 79.76
Tmax (mins.)	26.25 ± 6.71	30	18.75 ± 6.71*
Cmax (ng/ml)	116.94 ± 8.42	218.86 ± 15.73***	222.42 ± 21.24***
AUC _0–120_	9731.214 ± 621.1	15282.49 ± 1986.72***	20165.09 ± 813.73***
AUC _0–inf_	21370.60 ± 3776.34	29205.84 ± 15579.16	66503.206 ± 21035.76***
MRT	187.54 ± 52.73	143.44 ± 78.37	311.036 ± 115.29

*p < 0.05, **p < 0.01, ***p < 0.001 with respect to control group (B12 alone).

t1/2 (half- life), Cmax (maximum plasma concentration), Tmax (time at which maximum plasma concentration obtained), AUC_0–120_ (Area Under Curve from 0 to 120 min), AUC_0–inf_ (Area Under Curve from 0 to infinity), MRT (Mean Residence Time). Data expressed as mean ± SD (*n* = 5).

## Discussion

Different approaches are being adopted worldwide to prevent and treat vitamin B12 deficiency in individuals which include food fortification, oral supplementation, and intramuscular injections (23, 24). All these strategies intend to maintain optimal B12 levels in the body to avert and alleviate deficiency-associated symptoms. In a therapeutic regime, supplementation of B12 involves the administration of a high dose of Cyanocobalamin through oral route in the form of capsules/pills. Oral supplementation being more convenient and patient-friendly is preferred by individuals as compared to parenteral administration such as intramuscular injections of B12 (25). The overall goal of supplementation is to facilitate the restoration of micronutrients to normal levels in the body sufficient enough to mitigate deficiency conditions. Various strategies for the treatment of B12 deficiency through enhancing absorption by different methods have also been reviewed (26).

Bioavailability is a crucial parameter affecting micronutrient status inside the body. Deficiency of specific nutrients may also arise due to their low bioavailability. The traditional system of medicine relies on plants gifted by nature to address numerous health-related issues. To combat problems associated with low bioavailability, many herbal components have been recognized as bioenhancers which have shown promising results in augmenting the bioavailability of many pharmaceutical compounds. These bioenhancers have demonstrated bioavailability enhancing potential (upon co-administration with drugs) *in vitro* and also *in vivo* (in animal models and human subjects), they have been found to improve the bioactivity and pharmacokinetic profile of various categories of drugs (antibacterial, antifungal, anticancer, antiparasitic, immunosuppressants, etc.) (11). Most of the identified phytoconstituents have been found to be effective as bioenhancers of drugs and very few exist for micronutrients (27, 28). Furthermore, literature is inadequate with respect to research studies directed towards exploiting herbal plant extracts for increasing the oral bioavailability of Vitamin B12. Hence, this study was undertaken to evaluate the potential of ethanolic extract of *Glycyrrhiza glabra* to increase the bioavailability of Vitamin B12. GgEtOH was prepared from the roots of the plant. In the HPLC analysis of extract, some of the identified marker components included LTG, ISL, F, GA, and GLB. The effect of GgEtOH on stimulating intestinal absorption of vitamin B12 was investigated through *in vitro, ex vivo*, and *in vivo* assays. It was observed that GgEtOH increased the transport of B12 across Caco-2 monolayers and everted gut sacs which might be due to the extract components exhibiting permeation enhancing effect on the intestine membrane. Glycyrrhizic acid and its derivative Glycyrrhizin have been reported to modulate membrane permeability causing enhanced intestinal penetration of various drugs/bioactive compounds thereby leading to improvement in their bioavailability (29–32). Augmentation of systemic B12 levels *in vivo* could also be attributed to the possible prokinetic effect of extract (components) resulting in enhanced absorption of B12 inside the body. Studies have shown that extract of *G. glabra* and its constituent phytomolecule (Isoliquiretigenin) modulate gastric emptying and gastrointestinal transit of marker compounds/drugs (33, 34). The ileum is the primary site of B12 absorption in the small intestine and chronic inflammatory conditions (such as Crohn’s disease, Celiac disease, etc.) adversely affect its functioning consequently leading to inhibition of Vitamin B12 absorption (35–37). Hence, we also examined the anti-inflammatory effect of GgEtOH *in vitro* on RAW 264.7 cell line under LPS stimulation where we observed that GgEtOH at concentrations ranging from 0.1 to 100 μg/ml significantly suppressed the levels of pro-inflammatory cytokines (TNF-α and IL-6) *in vitro* (Supplementary file). This is consistent with the existing reports where *G. glabra* extract and its constituents have been found to perturb inflammation through different mechanisms in *in vitro* and *in vivo* models of inflammation (38). Hence, additionally, GgEtOH might assist in the absorption of B12 under inflammatory conditions inside the body by virtue of its anti-inflammatory property. Altogether, these observations indicate that the ethanolic extract of *Glycyrrhiza glabra* could enhance Vitamin B12 bioavailability by exerting influence over intestinal absorption and controlling inflammation.

## Conclusion

Preliminary findings of this study suggest that the ethanolic extract obtained from the roots of *G. glabra* holds potential to enhance the bioavailability of Vitamin B12 by facilitating its absorption inside the body as demonstrated by enhancement in intestinal permeation *in vitro* and *ex vivo* accompanied by the modulation of pharmacokinetic parameters *in vivo.* Oral administration of the extract in the form of a suitable herbal formulation may be a promising approach to improve Vitamin B12 status inside the body.

## Data availability statement

The raw data supporting the conclusions of this article will be made available by the authors, without undue reservation.

## Ethics statement

This animal study was reviewed and approved by the Committee for the Purpose of Supervision and Experimentation on Animals, Government of India. Protocol no. CIMAP/IAEC/2019-21/06.

## Author contributions

PS, PP, and VT performed the experiments and analyzed the data. FK, KS, MD, and AP analyzed the data. PS, MD, and AP conceived the idea and drafted the manuscript. All authors contributed to the article and approved the submitted version.
