# Supplementary material for: Investigation of the potential of Glycyrrhiza glabra as a bioavailability enhancer of Vitamin B12
Source: Front Nutr. 2022 Oct 28;9:1038902. doi: 10.3389/fnut.2022.1038902 (PMC9650095; doi:10.3389/fnut.2022.1038902)
Supplement: Supplementary file 1 [file Table_1.DOCX]

Supplementary Material

**Table 1.** Permeation coefficient (*Papp*) and fold enhancement values of Vitamin B12 during *in vitro* Caco-2 based Vitamin B12 permeation assay. Data expressed as mean ±SD (n=4)

|  | **Apparent Permeability (*Papp*) (cm/s) Mean± SD** | **Fold enhancement in Vitamin B12 transport** |
| --- | --- | --- |
| **B12 alone (200 µg/ml)** | **4.69×10^-6^± 0.93** | **-** |
| **B12+GgEtOH 25 µg/ml** | **1.13×10^-5^± 0.08 *** | **2.47 ± 0.46** |
| **B12+GgEtOH 50 µg/ml** | **1.71×10^-5^± 0.46 ***** | **3.68 ± 0.81** |
| **B12+GgEtOH 100 µg/ml** | **2.68×10^-5^± 0.29 ***** | **5.9 ± 1.58** |

* p < 0.05 ** p < 0.01 *** p < 0.001 significantly different from control (B12 alone)

**Table 2** Permeation coefficient (*Papp*) and fold enhancement values of Vitamin B12 during everted gut sac assay. Data expressed as mean± SD (n=4)

|  | **Apparent Permeability (***Papp* **(cm/s) mean**±**SD** | **Fold enhancement in Vitamin B12 transport** |
| --- | --- | --- |
| **B12 alone (100 µg/ml)** | **6.14×10^-6^±1.51** | **-** |
| **B12+GgEtOH 250 µg/ml** | **1.55×10^-5^±0.36 ***** | **2.63±1.02** |
| **B12+GgEtOH 500 µg/ml** | **2.14×10^-5^±0.13 ***** | **3.73±1.12** |

* p < 0.05 ** p < 0.01 *** p < 0.001 compared to control (B12 alone)

**MTT (3-(4,5-dimethylthiazolyl-2)-2,5-diphenyltetrazolium bromide) Assay**

MTT assay was carried out to assess the possible cytotoxicity of GgEtOH on the Caco-2 cell line. `Caco-2 cells were seeded in 96 wells plate at a density of 30,000 cells per well in 100 µl MEM. Cells were allowed to grow to confluence for 24 hours at 37ºC in 5% CO_2_. After 24 hours, the growth medium was discarded from the wells. Stock solution of GgEtOH was diluted in MEM and added to wells at different concentrations ranging from 10 µg/ml to 1000 µg/ml (10, 25, 50, 100, 200, 400, 800, 1000). Cells were incubated at 37ºC in 5% CO_2_ for 24 hours. Wells with confluent cells containing only MEM served as a negative control, whereas cells treated with 0.1% Triton X-100 served as the positive control (3). After 24 hours, medium from wells of 96 wells plate was aspirated and 100 µl MTT solution (0.5 mg/ml in PBS, pH 7.4) was added to each well followed by further incubation for 3 hours. After 3 hours, MTT solution was discarded from the wells and 100 µl DMSO was added to solubilize blue-colored formazan crystals. After 10 minutes, the absorbance of the samples was measured at 570 nm using a microplate reader. 620 nm was the reference wavelength. The cell viability (%) was calculated according to the following formula: (sample OD/control OD) × 100%. Cytotoxicity (%) was calculated as 100% - cell viability (%) followed by determination of the IC_50_ value.

**Results**

The cytotoxic effect of GgEtOH extract on Caco-2 cell line was determined by MTT assay. Caco-2 cells were treated with extract at 10, 25, 50,100, 200, 400, 800 and 1000 µg/ml concentrations for 24 hours. 0.1% Triton X-100 treated cells were used as positive control in the assay. As indicated in Fig. 2, the cell viability (%) of cells upon treatment with extract from 10-100 µg/ml did not exhibit any significant difference in comparison to the negative control. However, at concentrations exceeding 100 µg/ml, GgEtOH extract significantly affected the viability of cells compared to untreated cells (negative control) indicating an increase in the cytotoxic effect of extract with increasing concentrations. The IC_50_ value of GgEtOH was found to be 682.96 µg/ml. Hence, the extract at concentrations (25, 50, and 100 µg/ml) used in *in vitro* B12 permeability assay was non-cytotoxic to Caco-2 cells.


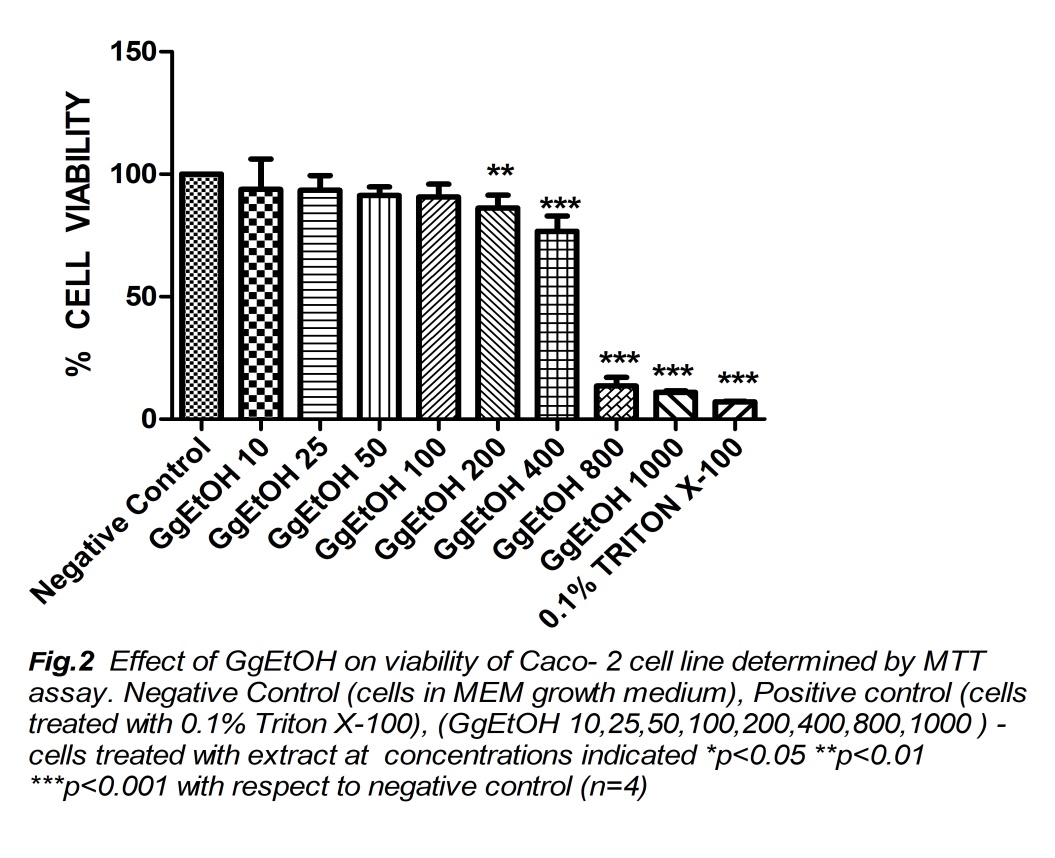


**Glucose transport assay**

To assess the viability/integrity of gut sacs during the ex vivo assay, glucose transport across the everted gut sacs (in all groups and time intervals) was measured (1). Glucose concentration was determined in mucosal and serosal fluid samples (10 µl) with the aid of a glucometer. S/M (Serosal to Mucosal ratio) was subsequently calculated.

**Result**

Tissue viability during everted gut assay was examined by determining the transport of glucose through sacs. Glucose is actively transported across biological membranes hence, in healthy (metabolically active) viable tissues, the concentration of glucose increases with time, whereas, in leaking damaged and non-viable tissues, such glucose transport is absent (2). Increasing serosal to mucosal ratio with time confirms the viability of tissues. Therefore, for the same reason, the concentration of glucose was measured in serosal (S) and mucosal (M) fluid/buffer samples of gut sacs in presence of Vitamin B12 (Cyanocobalamin) only and combination of Vitamin B12+GgEtOH (250, 500 µg/ml) at time intervals 30, 60, 90 and 120 minutes. It was found that the serosal to mucosal (S/M) concentration values of glucose in gut sacs in the B12 alone group was not significantly different from that of B12+GgEtOH 250 and B12+GgEtOH 500 groups during the entire two-hour duration (Fig. 4 & Table 3). This suggests that in presence of GgEtOH extract (at concentrations used), the viability of intestine/sac tissues was not compromised and the sacs were functioning properly.


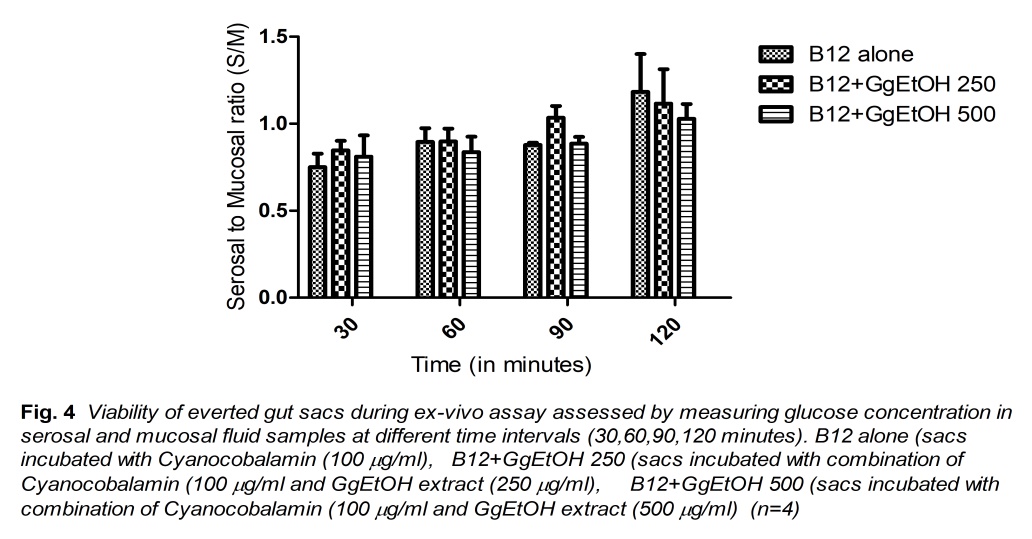


**Table 3** Serosal/Mucosal ratio (S/M) values of glucose obtained during everted gut sac assay. (mean± SD) (n=4)

| **Time (minutes)** | **B12 alone** | **B12+GgEtOH 250** | **B12+GgEtOH 500** |
| --- | --- | --- | --- |
| **30** | 0.75±0.078 | 0.85±0.06 | 0.81±0.12 |
| **60** | 0.895±0.08 | 0.897±0.07 | 0.84±0.09 |
| **90** | 0.88±0.01 | 1.03±0.064 | 0.89±0.04 |
| **120** | 1.18±0.22 | 1.12±0.19 | 1.03±0.085 |

**Anti-inflammatory activity of GgEtOH**

The anti-inflammatory property of ethanolic extract of *Glycyrrhiza* *glabra* (GgEtOH) was investigated *in vitro* on the murine macrophage cell line, RAW 264.7. Cells were seeded at a density of 1×10^5^ cells per well in 100 µl DMEM (supplemented with 10% FBS and antibiotics) in 96 wells plate and incubated at 37ºC in 5% CO_2_ for 24 hours to reach confluence. Cells were then treated with DMSO (vehicle control), Lipopolysaccharide (LPS-1 μg/ml conc.), and different concentrations of GgEtOH ranging from 0.1 to 100 μg/ml for 12-18 hrs. Cell supernatant was then collected for detection of pro-inflammatory cytokines (IL-6 and TNF-α) by ELISA (as per manufacturer’s instructions).

**Results**

The anti-inflammatory potential of ethanolic extract of *Glycyrrhiza glabra* was investigated in the murine macrophage cell line, RAW264.7. Cells were treated with DMSO (vehicle control), GgEtOH extract only (0.1,1,10,100 µg/ml), LPS only (1 µg/ml) and LPS+ GgEtOH (0.1,1,10,100 µg/ml). As shown in Fig.6 and Fig.7, inflammation was observed in cells in presence of LPS as indicated by an increase in levels of pro-inflammatory cytokines with concentrations of TNF-α and IL-6 exceeding 4000 pg/ml and 5000 pg/ml respectively. However, when LPS stimulated cells were treated with GgEtOH extract at 0.1,1,10,100 µg/ml concentrations, the levels of TNF-α and IL-6, normalized significantly to less than 1000 pg/ml. Increasing concentration of GgEtOH extract led to corresponding suppression in levels of TNF-α and IL-6 which was almost negligible at 100 µg/ml concentration of GgEtOH extract. GgEtOH extract in itself was inert, as it alone did not cause any inflammation in cells.


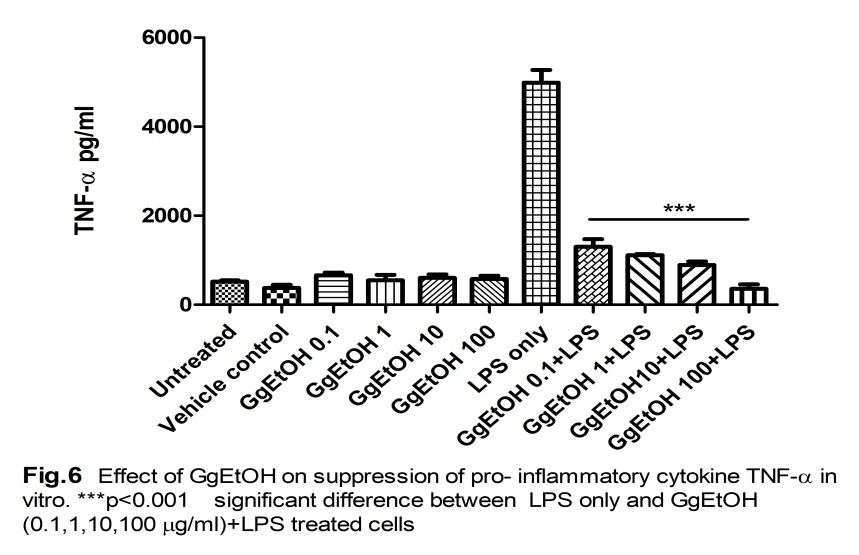


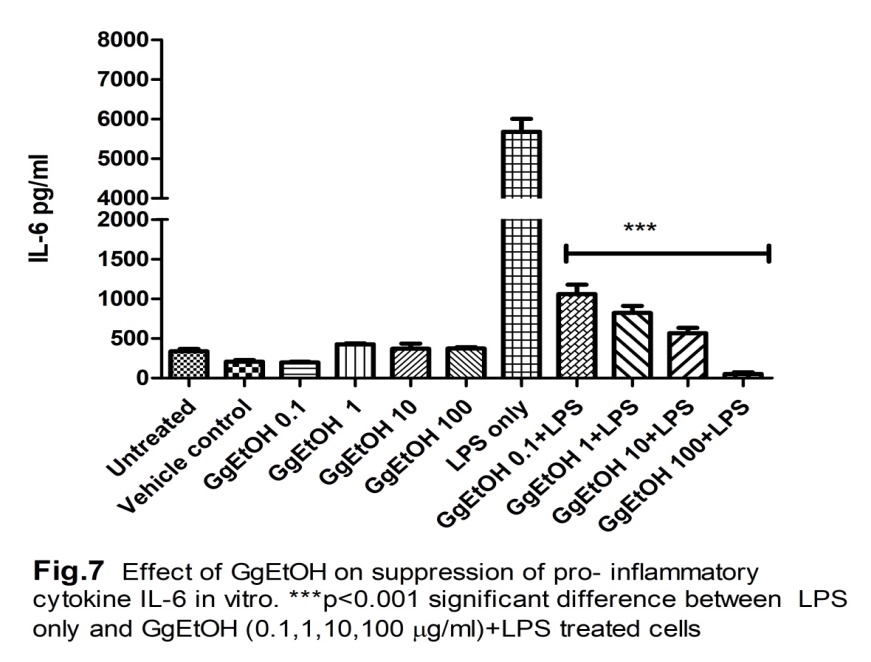


References

1. Hamilton KL, Butt AG. Glucose transport into everted sacs of the small intestine of mice. Adv Physiol Educ. 2013;37(4):415–26.doi.org/10.1152/advan.00017.2013
2. Barthe, L., Woodley, J.F., Kenworthy, S. et al. An improved everted gut sac as a simple and accurate technique to measure paracellular transport across the small intestine. European Journal of Drug Metabolism and Pharmacokinetics 23, 313–323 (1998). doi.org/10.1007/BF03189357
3. Netsomboon K, Feßler A, Erletz L, Prüfert F, Ruetz M, Kieninger C, et al. Vitamin B₁₂ and derivatives--In vitro permeation studies across Caco-2 cell monolayers and freshly excised rat intestinal mucosa. Int J Pharm. 2016;497(1–2):129–35.doi.org/10.1016/j.ijpharm.2015.11.043

**
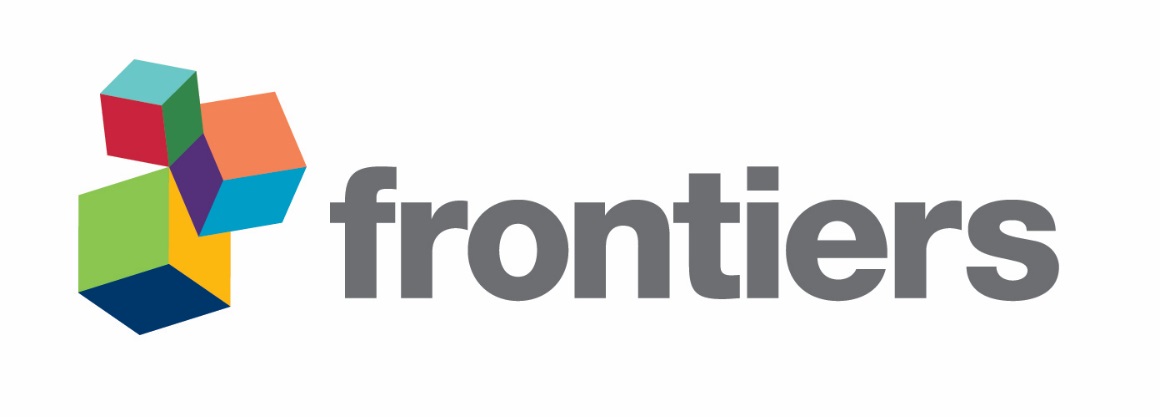
**
