# Supplementary material for: Investigation of the potential of Glycyrrhiza glabra as a bioavailability enhancer of Vitamin B12
Source: Front Nutr. 2022 Oct 28;9:1038902. doi: 10.3389/fnut.2022.1038902 (PMC9650095; doi:10.3389/fnut.2022.1038902)
Supplement: Supplementary file 2 [file Table_2.DOCX]

**Investigation of the potential of *Glycyrrhiza glabra* as bioavailability enhancer of Vitamin B12**

Vitamin B12 is involved in erythropoiesis and functioning of nervous and cardiovascular system of the body. Deficiency of Vitamin B12 is persistent among individuals globally. Insufficient dietary intake and low bioavailability are associated with the deficiency of B12. Approaches to overcome B12 deficiency include food fortification and supplementation, however, bioavailability remains a concern. Bioavailability of a nutrient refers to the fraction of an ingested nutrient that gets absorbed inside the body. Reduced nutrient bioavailability often leads to insufficient concentration of micronutrients inside the body which may also result in deficiency. Several herbal plant constituents have been identified as Bioavailability Enhancer of drugs. However, herbal extracts that can enhance Vitamin B12 bioavailability have hardly been investigated. *Glycyrrhiza glabra* is a herb with immense medicinal significance. Hence, we aimed to investigate the effect of ethanolic extract of *Glycyrrhiza glabra* on enhancing the bioavailability of Vitamin B12 through *in vitro, ex-vivo* and *in-vivo* assays. The extract enhanced B12 bioavailability as observed in *in vitro* (Caco-2), *ex vivo* (Everted gut sac) and *in vivo* (animal model) studies. These preliminary findings suggest that the ethanolic extract of *Glycyrrhiza glabra* can promote Vitamin B12 bioavailability which may help towards maintaining adequate B12 concentration inside the body.
